# Primary single suture anchor re-fixation of anterior cruciate ligament proximal avulsion tears leads to good functional mid-term results: a preliminary study in 12 patients

**DOI:** 10.1186/s13018-017-0678-9

**Published:** 2017-11-13

**Authors:** Christof Hoffmann, Jan Friederichs, Christian von Rüden, Christian Schaller, Volker Bühren, Christoph Moessmer

**Affiliations:** 1 Department of Trauma Surgery, BG Trauma Center Murnau, Professor Küntscher Str. 8, 82418 Murnau, Germany; 2 Department of Trauma Surgery and Sports Orthopaedics, Garmisch-Partenkirchen Medical Center, Garmisch-Partenkirchen, 82467 Germany; 3 Institute of Biomechanics, Paracelsus Medical University, Salzburg, Austria; 4 Department of Trauma Surgery and Orthopaedics, Brixen Medical Center, Brixen, Italy

**Keywords:** Anterior cruciate ligament (ACL), Single suture anchor re-fixation, Mid-term follow-up

## Abstract

**Background:**

Current studies demonstrate encouraging short-term results after primary anterior cruciate ligament (ACL) suture anchor repair. However, earlier studies reported deterioration of knee function at 5-year follow-up following good clinical short-term recovery. Therefore, the aim of this study was to evaluate clinical long-term results after primary ACL repair at a minimum 5-year follow-up.

**Methods:**

In a retrospective study, 13 patients were included between 2009 and 2012. Inclusion criteria were an acute proximal, femoral avulsion tear of the ACL with good tissue quality and sagittal instability in a healthy, demanding patient. Patients suffering proximal tibial fractures, arthrosis, or multiligamentous injuries of the knee were excluded. The ACL was anchored to the footprint by a single 2.9-mm push lock anchor, followed by additional microfracturing. For follow-up, patients were evaluated according to Lysholm score, modified Cincinnati score, and Tegner activity score. Clinical examination was performed using Lachman and pivot-shift testing and range of motion and sagittal stability measurement, using a Rolimeter.

**Results:**

Mean follow-up was 79 (range 60 to 98) months. One patient was lost to follow-up, and 11 out of 12 patients were examined clinically. Eight patients achieved good subjective and clinical outcome. One patient suffered an early re-tear, and one patient with additional patellar tendon tear and one patient with polyarthritis demonstrated poor subjective and clinical results due to lasting instability. Seven out of 12 patients reached preoperative Tegner activity score postoperatively again. The mean Lysholm score was 85.3 points, mean subjective IKDC score was 87.3 points, and mean modified Cincinnati score was 83.8 points. Rolimeter measurements demonstrated a mean side-to-side difference of 2 (range 1–5) mm.

**Conclusion:**

In the current study, primary surgical re-fixation of proximal, femoral ACL avulsion tears using single suture anchor repair resulted in good to excellent clinical mid-term outcomes. However, in cases of additional serious damage to extensor structures or systemic rheumatic disease, loss of function and unsatisfying clinical results occurred. Further prospective randomized controlled trials are necessary to confirm the encouraging long-term results of this study.

**Trial registration:**

Bavarian National Medical Chamber of Physicians, file number 2016-095. German Clinical Trials (DRKS00013059)

## Background

Primary ACL repair has been discussed controversially in the past. Although early attempts with open ACL repair led to promising short-term results, mid- to long-term results [1, 2] appeared to be disappointing. In consequence, ACL primary repair was largely abandoned and ACL reconstruction was established as the current standard of treatment. However, early open ACL repair techniques [1] were followed by long lasting cast immobilization and partial weight bearing. Suture repair was performed via arthrotomy, drill holes and regardless of tissue quality, tear-type, and concomitant injuries. Since then, both, operation techniques as well as rehabilitation protocols, have been developed.

As ACL reconstruction may be associated with comorbidities such as donor site morbidity, physeal disruption, and loss of proprioceptive properties [3], the idea of ACL preservation was reconsidered again since ACL primary repair represents a less invasive procedure.

Besides, classifying ACL ruptures regarding type of tear and tissue quality, Sherman et al. reported in 1991 that a subset of patients with proximal avulsion tears of the ACL was prone to have better functional outcomes [4]. Based on this study, DiFelice et al. and Achtnich et al. introduced techniques of arthroscopic ACL preservation via suture anchor re-fixation and reported excellent subjective and clinical results [3, 5]. Achtnich et al. even found that primary repair restores knee stability comparable with single bundle ACL reconstruction [5]. Both studies demonstrated short- to mid-term results after mean follow-up periods of 41 and 28 months.

But concerns about long-term results still remained as presented in recent literature: Knee function may deteriorate to poor outcome [6], even if short-term results are promising [7]. Deterioration was marked at a follow-up of 5 years.

To the best of our knowledge, the current clinical trial represents the first study in English literature to report mid-term results following one-staged single suture anchor repair of proximal ACL avulsion tears with a minimum follow-up of 5 years. We hypothesize that clinical outcomes will remain good and that no loss of function or stability will occur.

## Methods

A retrospective clinical trial including 13 consecutive patients with ACL knotless suture anchor re-fixation technique between 2009 and 2012 was performed. Surgical interventions were performed by a team of two senior physicians. ACL re-fixation was indicated in all cases in accordance with preoperative clinical and radiological evaluation and intraoperative assessment of the ligament stability. ACL primary repair was performed only in case of type 1 true soft tissue avulsions without any sign of interstitial tearing and intact synovial coverage of the ligament. In case of inadequate tissue quality, operation was converted to ACL reconstruction. Further inclusion criteria were unilateral acute ACL ruptures, absence of concurrent contralateral knee injuries, a grade 2 to 3 sagittal instability in Lachman testing, and a healthy, in terms of sports activities, demanding patient. Patients were excluded in case of significant arthrosis and multiligamentous injuries around the knee (> 3 ligaments). There were no age restrictions.

All patients were evaluated according to modified Cincinnati score, Tegner activity score, Lysholm score, and Subjective International Knee Documentation Committee (IKDC) score. Anterior knee stability was measured by the Rolimeter (Aircast, DonJoy, Freiburg, Germany) by two different examiners who were blinded to this study.

### Surgical technique

**Fig. 1:**
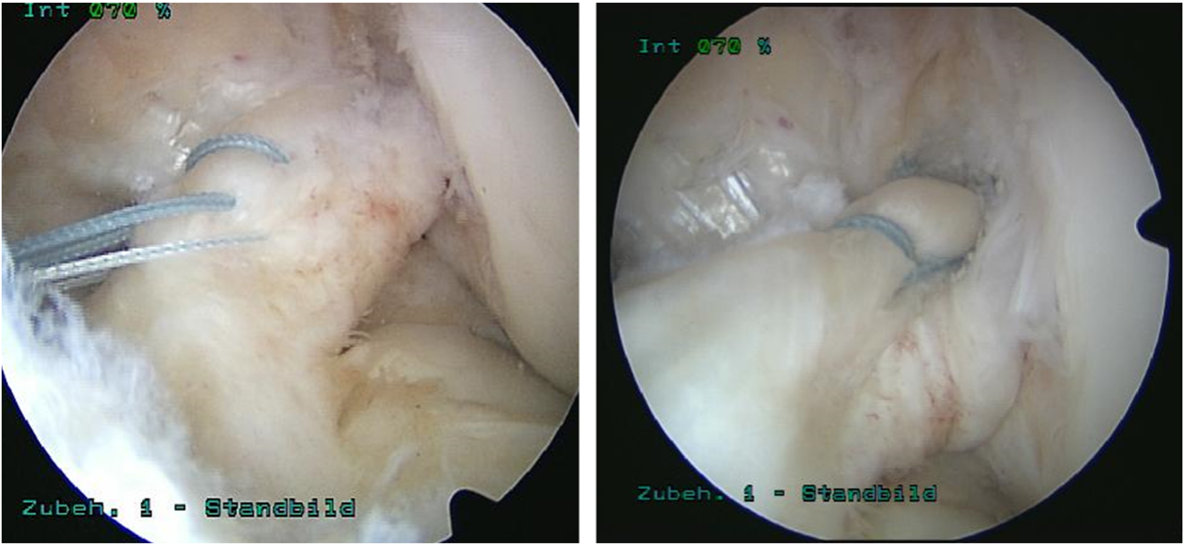
Arthroscopic view of a left knee with proximal avulsion tear of the ACL. The antero-medial bundle is mounted utilizing a no. 2 Fiber Wire, the postero-lateral bundle using a no. 0 Fiber Wire. The image was taken before the second stich through the postero-lateral bundle. Image no. 2 shows the final result after femoral ACL re-fixation

The patient was placed in supine position, and the leg was prepared in standard fashion. A high anterolateral portal was created, and diagnostic arthroscopy was performed routinely. After creating an additional anteromedial portal, the ligament was assessed by probing under direct vision. Concomitant injuries were treated first. After diagnosis of a proximal avulsion tear Sherman type 1 [4] with excellent tissue quality, a number 2 Fiber Wire® (Arthrex, Naples, FL, USA) was passed twice through the anteromedial bundle of the ACL using a Scorpion suture passer in 40° knee flexion through the anteromedial portal. No cannulas were used. The first suture was placed in the middle third of the ligament. The second stich was placed in the proximal third of the ligament remnant. Afterwards, the procedure was repeated using a number 0 Fiber Wire® at the proximal and middle third of the posterolateral bundle (Fig. 1). Special care was taken to rule out soft tissue interposition at the anteromedial portal or a miss-link between the two sutures. The suture tails were threaded through the eyelet of a 2.9-mm push lock anchor. With the knee flexed to 90°, a 2.9 × 20 mm hole was drilled into the origin of the ACL footprint under direct visualization. While the anchor was put into the drill hole, the wire limbs were released when the first rim of the anchor was covered by bony cortex. The anchor was forwarded into the socked to the second laser line. After removing the driver, the free ends were cut with an open-end suture cutter (Arthrex, Naples, FL, USA). Tension and stiffness were tested with a probe in 60° of knee flexion. Impingement was ruled out arthroscopically. Two to three microperforation holes were put into the cortex of the lateral femoral condyle with an awl surrounding the ACL. No drainage was utilized.

### Aftercare scheme

Postoperative care included partial weight bearing for 2 weeks, a knee brace with full extension and flexion limitation to 90° for 6 weeks, control of swelling, and early gain of range of motion. In case of meniscal repair, flexion was limited to 60° for 4 weeks and to 90° for additional 2 weeks. For the time of inpatient treatment, continuous passive motion (CPM) was implemented. After 6 weeks, the patients weaned off the brace and started muscle strengthening according to a standard ACL rehabilitation protocol.

## Results

Between 2009 and 2012, 13 patients (4 male, 9 female) underwent primary ACL suture anchor repair. The mean age at day of surgery was 43.3 (range 19–67) years. Nine patients had concomitant injuries including medial or lateral meniscus tears, tears of the medial or lateral collateral ligament (MCL/LCL), or chondromalacia. In one case, a bony avulsion of the LCL on the femoral side was treated by screw fixation; in one case, a tibial avulsion of the MCL was treated by cork screw re-fixation (Arthrex, Naples, FL, USA). The vast majority of patients (10 patients) suffered knee injury during sports activities (alpine and Nordic skiing, soccer, running), and 2 patients suffered knee damage during work. The mean delay from injury to surgery was 5.8 (range 1–20) days.

One patient suffered additional patellar tendon rupture and was treated using additional patellar tendon reconstruction including McLaughlin cerclage for 3 months. This patient received a rehabilitation protocol including a brace locked in full extension for 2 weeks and subsequent release of knee flexion from 30° to 60° to 90° over 6 weeks. Partial weight bearing was claimed for 6 weeks, then full weight bearing with the knee fully extended was allowed. The cerclage was removed after 3 months.

One patient reported a history of polyarthritis, but no signs of ongoing inflammation were found during the operation. Postoperative complications, such as infection or impairment of wound healing, were not observed. All patient characteristics are summarized in Table 1.

**Table 1:** Demographic characteristics, concomitant injuries

	Gender	Age (years)	Follow-up (months)	Mechanism of injury	Concomitant injuries/pathologies
Patient 1	Male	43	98	Alpine skiing	°II CM
Patient 2	Male	26	97	Bob sleight	
Patient 3	Female	34	96	Alpine skiing	
Patient 4	Female	62	86	Nordic skiing	MCL rupture, medial meniscus tear, °II CM
Patient 5	Male	19	77	Soccer	Medial meniscus tear
Patient 6	Female	67	75	Hiking	MCL injury
Patient 7	Male	39	75	Alpine skiing	Patellar tendon tear
Patient 8	Female	45	74	Alpine skiing	Femoral LCL avulsion, lat. meniscus tear
Patient 9	Female	43	73	Work accident, walking	Medial meniscus tear, polyarthritis
Patient 10	Female	47	71	Alpine skiing	Medial meniscus tear, °II CM
Patient 11	Female	51	61	Alpine skiing	Medial meniscus tear
Patient 12	Female	45	60	Work accident, walking	
Mean ± SD		43 ± 13	79 ± 13		

**Fig. 2:**
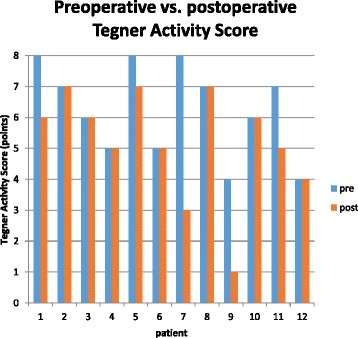
Mid-term results of postoperative Tegner activity score compared to preoperative activity level

**Fig. 3:**
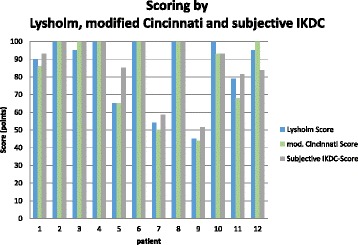
Mid-term functional outcome according to Lysholm, modified Cincinnati, and subjective IKDC scores

After a mean follow-up of 79 (range 60–98) months, 12 patients were evaluated according to clinical scores. One female patient was lost to follow-up. Eleven patients were clinically examined; 1 patient was evaluated via a telephone interview and sent his filled scores back. The mean Lysholm score was 85.3 points, and the mean modified Cincinnati score was 83.8 points. The subjective IKDC score was 87.3 points, the objective IKDC score was A in 8 patients, B in 1 patient, and C in 2 patients. In two cases, satisfaction and subjective scores demonstrated poor results. Rolimeter assessment demonstrated a side to side difference less than 3 mm in 9 patients, while in 2 patients the difference was 4 to 5 mm. Follow-up results are shown in Table 2 and Figs. 2 and 3.

**Table 2:** Patients’ data, functional scores, clinical examination, and Rolimeter testing

	Lysholm score	Tegner activity score		Modified Cincinnati score	Subjective IKDC score	IKDC physical examination	Differences in Rolimeter testing	Giving way	Duration of work incapacity	Type of work
		pre	post				30° knee flexion/mm		(weeks)	
Patient 1	90	8	6	86	93.1			pos 1/month	2	Office
Patient 2	100	7	7	100	100	A	2	neg	4	Office
Patient 3	95	6	6	100	100	A	1	neg	4	Office
Patient 4	100	5	5	100	100	A	2	neg	6	Office
Patient 5	65	8	7	79	85.1	B	2	neg	3	Electrician
Patient 6	100	5	5	100	100	A	1	neg	6	Retiree
Patient 7	54	8	3	50	58.6	C	4	pos 2/month	12	Office
Patient 8	100	7	7	100	100	A	1	neg	6	Office
Patient 9	45	4	1	44	51.7	C	5	pos 1/month	12	Farmer
Patient 10	100	6	6	93	93.1	A	1	neg	6	Office
Patient 11	79	7	5	68	81.6	A	1	neg	8	Workman
Patient 12	95	4	4	100	83.9	A	2	neg	6	Farmer
Mean	85.3	6.3	5.2	83.8	87.3		2.11		6.3	
± SD	19.9	1.5	1.8	21.3	16.6				3.1

The results of three patients had to be considered failures: One patient with the additional patellar tendon tear needed revision surgery due to a lasting feeling of instability. No subsequent trauma took place in the meantime. The surgical report from revision surgery, performed 2 years after the initial operation in another hospital, noted a “complete loss of the ACL.” Revision ACL reconstruction was performed using a semitendinosus tendon.

One patient experienced an atraumatic pop during a clinical follow-up examination according to the Lachman test 8 weeks after surgery. Delayed MRI imaging 4 weeks after the injury secured a re-tear of the ACL. The patient was treated conservatively, and mild instability developed. So far, the patient rejects treatment with ACL reconstruction.

One patient with a history of rheumatoid arthritis developed a persistent subjective feeling of instability 2 years after the operation. An infestation of polyarthritis of the concerning knee joint was denied at presentation. A MRI revealed a complete loss of the ACL remnant. The patient rejected to receive revision surgery due to bad experiences with ACL reconstruction surgery at the contralateral knee joint 4 years prior to the accident. Arthrofibrosis and lasting knee pain had led to five-time revision surgery after ACL reconstruction with semitendinosus tendon. All three patients reported a feeling of giving way. Lachman and pivot-shift testing demonstrated grade 1 to 2 instability in two out of those three patients. Despite ACL reconstruction surgery, the patient with the additional patellar tendon tear retained subjective knee instability and did not recover to preoperative activity level.

## Discussion

In this study, 12 cases of primary suture anchor re-fixation of proximal avulsion tears of the ACL have been reviewed. A single suture anchor re-fixation including both bundles has been performed. Excellent subjective and clinical results have been evaluated in 7 patients and mild impairment of knee function in 2 patients after a minimum follow-up of 5 years. One patient reported early re-tear 8 weeks after surgery, and 2 patients complained about constant, impairing knee instability. There were no postoperative complications and no delayed deterioration of knee function found in our cases. Seven patients (58%) demonstrated excellent knee function without any limitations in sports or activities of daily life. The mean Lysholm Score of this subpopulation was 98.6 points. All these patients recovered completely to their preoperative sport level and were able to maintain this level for 5 years. Rolimeter assessment of sagittal stability in 5 out of these 7 cases demonstrated excellent stability. Two patients reported mild impairment of knee function with recurrent swelling after extensive sport activities. One out of these patients had to modify her sports activities and demonstrated a decrease of 2 points in Tegner activity score due to swelling and mild pain but without instability. One patient complained about a slight feeling of instability, but he reported this feeling in both knees. He was able to return to contact sports with only slight limitations and a decrease of 1 point in Tegner activity score. The mean Lysholm score of these two patients was 79.5 points; Rolimeter testing demonstrated a mean side-to-side difference of 1.5 mm.

### Potential reasons for impaired g-term functional results

However, three patients (25%) reported lasting impairment of knee function including instability in two cases and one early re-tear during clinical examination. One must assume that the ACL did not heal properly in those three cases.

The patient with the early re-tear during clinical examination was the very first patient who underwent ACL re-fixation in our department. The re-tear happened with a pop sensation during clinical investigation 8 weeks after the index procedure. The load on the ACL was inadequate which suggests the absence of healing of the ACL. However, early re-tears also occur in recent studies published by Achtnich et al. and DiFelice et al. [3, 5] Both, Achtnich and DiFelice traced their failures back to noncompliant patients with refusal of adequate postoperative treatment, no sticking to the rehabilitation protocol, or too early return to sports. In contrast, our patient stuck to the postoperative rehabilitation protocol.

### Special case: simultaneous traumatic patellar tendon rupture and ACL rupture

One other patient suffered from severe knee distortion with a concomitant patellar tendon tear. This patient underwent revision surgery 2 years after primary surgery due to a persisting feeling of instability. The surgical report described a complete loss of the ACL fibers. The patient’s subjective knee function did not improve after ACL single bundle reconstruction. Due to persistent pain, he had to quit sports completely and still requires sporadic pain medication. Simultaneous traumatic patellar tendon rupture and ACL rupture is an uncommon injury and is rarely reported in literature [8]. No clear treatment regime has been established yet. Nevertheless, there is an agreement that immediate repair of the patellar tendon is recommended [9, 10]. Two-staged surgical treatment is preferred by a few authors [11, 12], but also one-staged procedures are reported to lead to satisfying functional results [10, 13, 14]. In this case, a one-staged surgical approach with primary suture anchor repair of the ACL and suture of the patellar tendon competes with a single repair of the patellar tendon and ACL reconstruction in a second step. Although in our case the ACL did not heal, there were no signs for intraoperative problems caused by the enclosed push lock anchor in the surgical report of ACL reconstruction. In contrary to preceding studies, in our case, no extravasation of fluid during arthroscopy occurred. In the described patient, not stiffness or arthrofibrosis [10, 13], but recurrent instability and lasting pain were attributed to be the main problem. Retrospectively, severe damage to the knee extensor structures seemed to impede adequate healing of the ACL fibers.

### Surgical technique

The surgical technique of ACL re-fixation is still subject of controversial discussion. However, based on current literature reports, there is no clinical evidence for supremacy of one or another technique. Achtnich et al. introduced a single-bundle re-fixation whereas DiFelice et al. and Weninger et al. performed a double-bundle re-fixation with two anchors [3, 5, 15]. Achtnich et al. combined a direct suture anchor re-fixation with the healing response technique and reported early re-tear in one case and recurrent instability in two cases [5]. DiFelice reported one clinical failure with early re-tear after 3 months [3]. We believe that one advantage of a single anchor re-fixation, in addition to a reduced amount of suture material placed in the ACL, might be reduced foreign material in the femur. In case of revision surgery, the anchors are supposed to be over drilled to create an adequate bony socket. Parts of the anchors might get spread through the joint by drilling, and osseous defects of the femoral insertion zone might occur. However, no intraoperative problems associated with the enclosed anchors have been reported in the described studies, which underlines, that ACL reconstruction after primary suture anchor repair is comparable with primary reconstruction.

The abandonment of ACL primary suture anchor repair can be traced back to the landmark studies of Feagin et al. and Taylor et al. reporting open suture repair of ruptured ACL via arthrotomy [1, 7]. In their studies of West Point Cadets, Feagin et al. reported an instability rate of 94%, pain and stiffness rate of 71%, and swelling rate of 66% at a 5-year follow-up despite initially promising short-term results.

### Results of this study in context of current literature

**Fig. 4:**
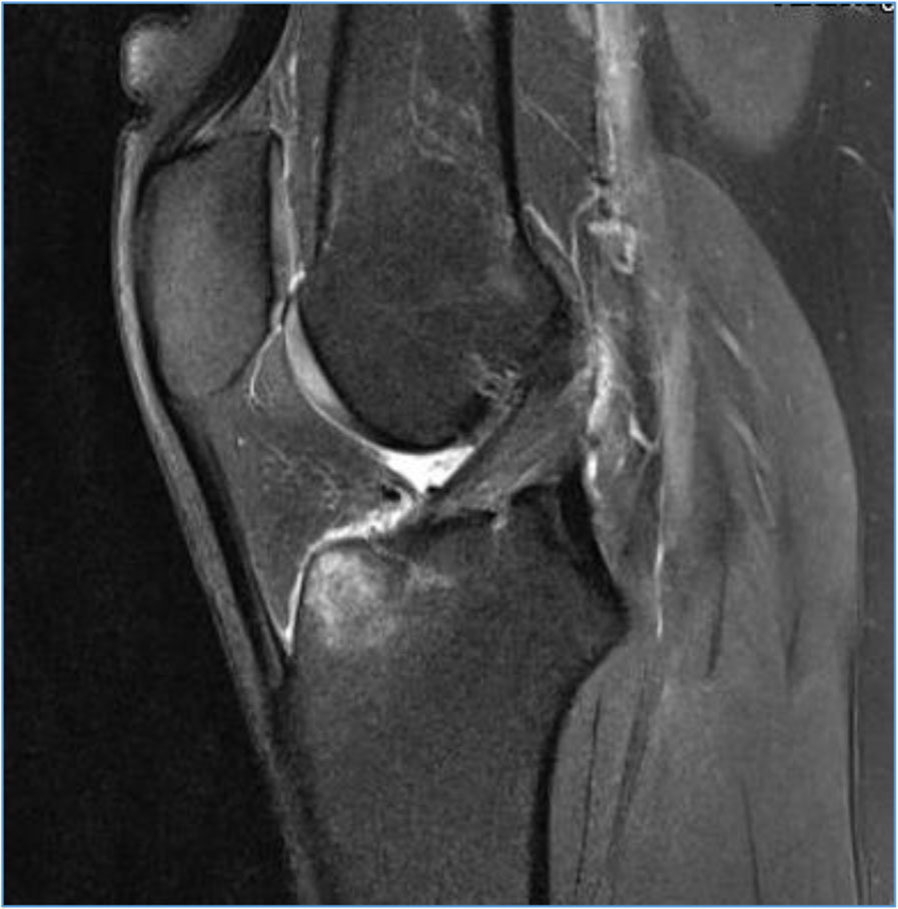
MRI scan of a 26-year-old male patient 2 years after primary arthroscopic suture anchor re-fixation of the ACL. The patient presented again in terms of a second injury including hyperextension of the knee. Up to this second injury, the patient was pain free and fully recovered to his preoperative sports level

In our investigation, we could not find any delayed deterioration of knee function. All cases which had considered to be failures demonstrated unsatisfying knee function from the beginning. The one incident of atraumatic re-tear occurred 8 weeks after surgery, and the instability in two other cases appeared immediately after the index procedure. Taylor et al. reported in their long-term follow-up after 30 years that 64% of their patients had at least one more knee surgery, in 28% due to instability. Fifty percent of those patients recovered well. In our cohort, one patient needed revision surgery, and one patient rejected revision surgery despite instability. In addition, Taylor et al. reported that the 5-year results were supposed to be a good predictor of the subjective results after more than 30 years. This generates expectations that even mid-term results following primary suture anchor repair remain good or excellent. In addition, follow-up MRI scans or second look arthroscopies could highlight the status of the ACL remnant also in clinically asymptomatic patients (Fig. 4).

### Limitations

In addition to the small size of our study cohort, the limitations of this study include the variety of age of patients, of patients’ preoperative activity level (Tegner activity score preoperatively 4–8 points), and the relatively heterogeneous nature of the cohort group. Furthermore, we report on surgical results of two different senior surgeons which may have influenced the results.

## Conclusions

In the current study, primary one-staged surgical re-fixation of proximal, femoral ACL avulsion tears using single suture anchor repair technique resulted in good to excellent functional mid-term outcomes. Serious damage to extensor structures and systemic rheumatic diseases may lead to a loss of function and unsatisfying clinical results. So, this technique should only be used in carefully selected patients. Further prospective randomized controlled trials are mandatory to confirm the encouraging mid-term results provided by this study.

## Abbreviations

ACL: Anterior cruciate ligament
CM: Chondromalacia
CPM: Continuous passive motion
IKDC: International Knee Documentation Committee
LCL: Lateral collateral ligament
MCL: Medial collateral ligament
MRI: Magnet resonance imaging
SD: Standard deviation

## References

[1] Taylor DC, Posner M, Curl WW, Feagin JA (2009). Isolated tears of the anterior cruciate ligament: over 30 year follow-up of patients treated with arthrotomy and primary repair. Am J Sports Med.

[2] Strand T, Molster A, Hordvik M, Krukhaug Y (2005). Long-term follow up after primary repair of the anterior cruciate ligament: clinical and radiological evaluation 15-23 years postoperatively. Arch Orthop Trauma Surg.

[3] DiFelice GS, Villegas C, Taylor S (2015). Anterior cruciate ligament preservation: early results of a novel arthroscopic technique for suture anchor primary anterior cruciate ligament repair. Arthroscopy.

[4] Sherman MF, Lieber L, Bonamo JR, Podesta L, Reiter I (1991). The long-term follow up of primary anterior cruciate ligament repair. Defining a rationale for augmentation. Am J Sports Med.

[5] Achtnich A, Herbst E, Forkel P, Metzlaff S, Sprenker F, Imhoff AB, Petersen W (2016). Acute proximal anterior cruciate ligament tears: outcomes after arthroscopic suture anchor repair versus anatomic single-bundle reconstruction. Arthroscopy.

[6] Taylor SA, Khair MM, Roberts TR, DiFelice GS (2015). Primary repair of the anterior cruciate ligament: a systematic review. Arthroscopy.

[7] Feagin JA, Curl WW (1976). Isolated tear of the anterior cruciate ligament: 5-year follow up study. Am J Sports Med.

[8] Chow FY, Wun YC, Chow YY (2006). Simultaneous rupture of the patellar tendon and the anterior cruciate ligament: a case report and literature review. Knee Surg Sports Traumatol Arthrosc.

[9] Rae PJ, Davies DR (1991). Simultaneous rupture of the ligamentum patellae, medial collateral, and anterior cruciate ligaments. A case report. Am J Sports Med.

[10] Chiang AS, Shin SS, Jazrawi LM, Rose DJ (2005). Simultaneous ipsilateral ruptures of the anterior cruciate ligament and patellar tendon: a case report. Bull Hosp Jt Dis.

[11] Koukoulias NE, Koumis P, Papadopoulos A, Kyparlis D, Papastergiou SG (2011). Acute, simultaneous tear of patellar tendon and ACL: possible mechanism of injury and rationality of the two-stage surgical treatment. BMJ Case Rep.

[12] Achkoun A, Houjairi K, Quahtan O, Hassoun J, Arssi M, Rahmi M, Garch A (2016). Simultaneous rupture of the anterior cruciate ligament and the patellar tendon: a case report. Pan Afr Med J.

[13] Futch LA, Garth WP, Folsom GJ, Ogard WK (2007). Acute rupture of the anterior cruciate ligament and patellar tendon in collegiate athlete. Arthroscopy.

[14] Cucchi D, Aliprandi A, Nocerino E, Randelli P. Early combined arthroscopic treatment for simultaneous ruptures of the patellar tendon and the anterior cruciate ligament leads to good radiological results and patient satisfaction. Knee Surg Sports Traumatol Arthrosc. 2017 Apr 29. doi:10.1007/s00167-017-4562-2. [Epub ahead of print]. 10.1007/s00167-017-4562-2 28456816

[15] Weninger P, Wepner F, Kissler F, Enenkel M, Wurnig C (2015). Anatomic double-bundle reinsertion after acute proximal anterior cruciate ligament injury using knotless pushLock anchors. Arthrosc Tech.

